# InNMR: Direct *In Situ* Studies of Transport Phenomena Enabled by an Innovative NMR-Tube Insert

**DOI:** 10.1021/acs.analchem.6c01297

**Published:** 2026-05-25

**Authors:** Amelie Frison, Marit M. Sørensen, Kristoffer Prince, Maksim Mayzel, Finn. L. Aachmann, Gøril Eide Flaten, Philip Rainsford, Johan Isaksson

**Affiliations:** † Department of Pharmacy, 8016 UiT the Arctic University of Norway, Box 6050, Langnes, 9037 Tromsø, Norway; ‡ 206144 Bruker Switzerland AG, Industriestrasse 26, 8117 Fällanden, Switzerland; § Department of Biotechnology and Food Science, Norwegian University of Science and Technology (NTNU), Sem Sæland 6/8, 7491 Trondheim, Norway; ∥ Department of Chemistry, UiT the Arctic University of Norway, Box 6050, Langnes, 9037 Tromsø, Norway

## Abstract

A novel discoid insert that can be positioned into a standard NMR tube using an associated plunger tool has been developed. By attaching any type of semipermeable barrier to the bottom of the insert, the detection volume of the tube can be divided into two or more chambers, separated by a barrier of choice. Slice-selective experiments are then utilized to study a plethora of transport phenomena in real time by NMR spectroscopy. The technological device is presented together with a proof of concept for three different applications: permeability studies, diffusion-controlled pH titrations, and diffusion-controlled protein titrations. The developed approach unlocks new tools for scientists working in the fields of drug discovery, organic chemistry, biochemistry, chemical biology, microbiology, or electrochemistry that will enable new types of experiments to be performed by NMR spectroscopy.

## Graphical abstract

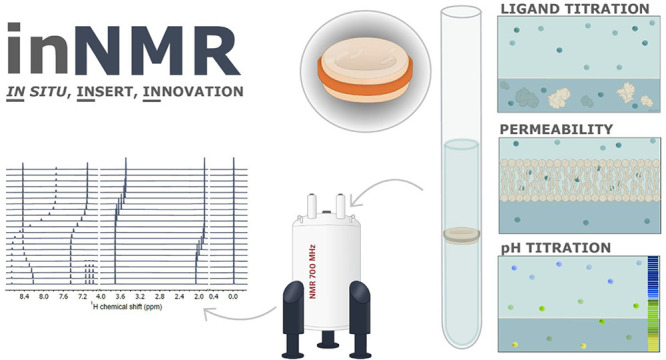

## Introduction

Nuclear magnetic resonance (NMR) has a rich history of innovations in tube design, unique experiment design, and various accessories to access new experimental parameters. Such developments include stretching gels and compression apparatus to extract dipolar couplings in liquid NMR,^1^ flow systems for reaction monitoring^2^ or separation by in-line liquid chromatography,^3^ and hyperpolarization in benchtop spectrometers to enhance sensitivity.^4,5^ The capacity of NMR to quantify individual components in multiple states or short-lived intermediate states^6,7^ has led to the development and adoption of *in situ* NMR methods with broad applications in battery and materials research and reaction monitoring.^8,9^ Through these time-resolved *in situ* NMR methods, mechanistic insights that cannot be easily accessed by other methods can be uncovered, enabling detailed models of battery processes,^10,11^ reaction mechanisms,^12^ and biological events^13^ to be built. *In situ* NMR methods have thus emerged as powerful cross-disciplinary tools.

Previous work with permeability models for drug delivery and absorption from our lab has identified NMR as a powerful detector for permeability assays.^14^ NMR is inherently quantitative and capable of detecting multiple atomic nuclei. On the basis of this, we have developed a simple and flexible insert that can separate a standard 5 mm NMR tube into two distinct volumes separated by a semipermeable barrier.^15^ By positioning the insert inside the detection volume of the sample, it is possible to use spatially resolved NMR spectroscopy to individually observe the two volumes in real time. This enables a plethora of experiments that involve transport across any type of semipermeable barrier to be performed.

The novel insert presented herein consists of a disk-based design that can be positioned at any height within a standard NMR tube through friction (Figure 1a) by using a specially designed plunger tool. The insert disk, to which many types of barriers can be glued, creates a watertight seal with an O-ring. This allows the same spectral quality and sensitivity to be achieved in both chambers. The insert enables a new class of diffusion-controlled titration experiments (Figure 1b) with minimal expense. Herein, we describe the details of the insert and demonstrate a proof of principle for three different potential applications: permeability, diffusion-controlled pH titration, and diffusion-controlled titration experiments of ligand–protein interactions.

**Figure 1:**
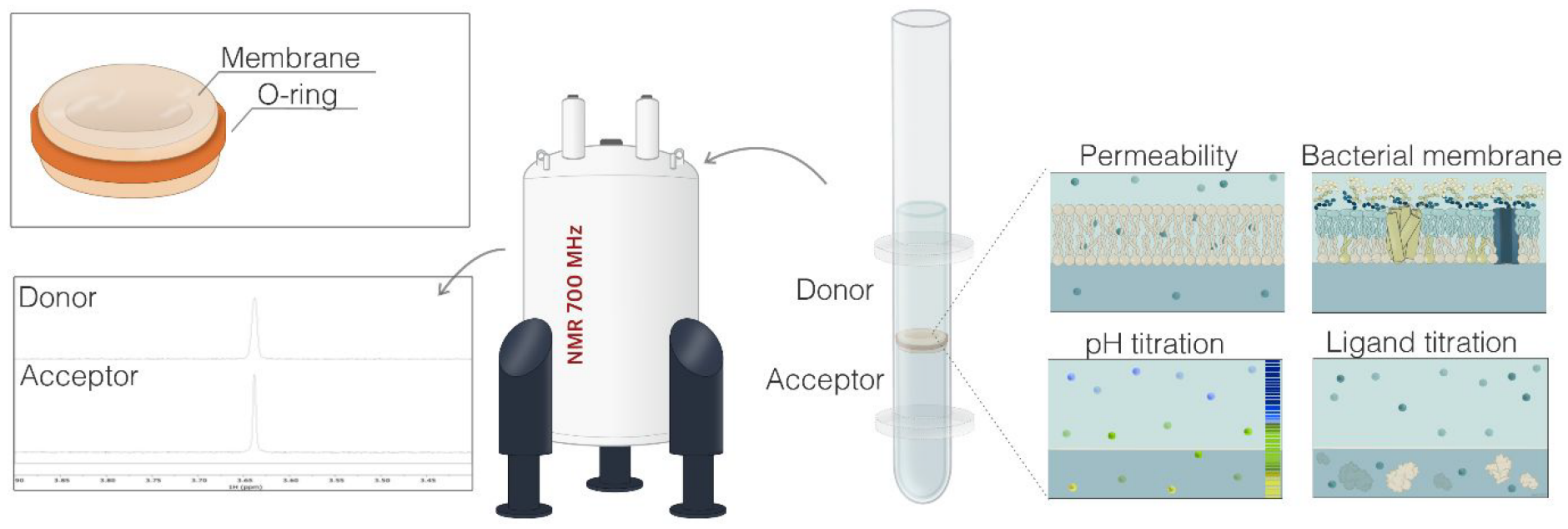
Schematic figure showing the insert disk with a membrane and potential applications for an NMR insert.

## Experimental Section

### Materials

Caffeine, terbutaline, imidazole, tris­(hydroxymethyl)­aminomethane (Tris), acetic acid, pyridine, terbutaline, 2,2-dimethyl-2-silapentane-5-sulfonate (DSS), 3-(trimethylsilyl)­propionic-2,2,3,3-*d*_4_ acid sodium salt (TMSP), and dodecane were purchased from Sigma-Aldrich. Ammonium formate was purchased from VWR. Sodium chloride was purchased from Riedel-de Haën. Egg phospholipids were purchased from Lipoid. All of these products were used as received without further purification.

### NMR Spectroscopy

NMR experiments were acquired on a Bruker Neo spectrometer equipped with an inverse triple-resonance TCI probe with cryogenic enhancement for ^1^H, ^13^C, and ^2^H, operating at 700 MHz for ^1^H. All experiments were performed at 298 K. All spectra were acquired on nonspinning samples. When using the insert disk inside the detection volume, the samples were first calibrated filled with only the acceptor solution, using standard procedures. The disk was then inserted into the center of the detection volume, and the calibrations were again touched up using only manual shimming of the low-order shims, before the top solution was replaced by the donor solution and acquisition was immediately started.

A double pulse field gradient spin echo (DPFGSE) type pulse sequence was used to acquire slice-selective spectra, where the selective inversion is made under a gradient for slice selection (slicesel_zgse in Figure S1a). The experiment was written with the option to use either the double echo or the single echo. For most samples studied with inserts placed inside the detection volume, the double echo is necessary for a satisfactory line shape. However, the double echo results in a longer sequence, and hence more pronounced *J* modulation. Therefore, for sample preparations in which this is not necessary, the single-echo variant results in cleaner multiplets. For nondeuterated samples, a slice-selective excitation with an excitation sculpting sequence was acquired, placing the excitation sculpting block after the slice selection block (slicesel_zges in Figure S1b). The pulse programs are provided in the Supporting Information and incorporate code for automatic calculation of the selective reburp/eburp pulses and offset corresponding to the desired slice height and position in millimeters. This requires accurate calibration of the gradient strength and the 90° proton pulse. It is possible to include perfect echo into these pulse sequences for improved line shapes.

### PAMPA

To establish the PAMPA barrier, a lipid solution was first prepared by dissolving E80 lipids (20% w/v) in dodecane. A thin layer of the solution was then applied to a Durapore 0.22 μM PVDF filter previously glued on the insert disk.

The tube was first filled with 600 μL of acceptor buffer (3.0 mM Tris and 2.3 mM TMSP in D_2_O (pH 7.4)). The PAMPA-mounted insert was positioned in the center of the detection volume. The buffer above the PAMPA barrier was removed, and its volume was precisely measured to determine the acceptor chamber volume (i.e., the solution remaining below the PAMPA barrier). Then, 300 μL of a ca. 10 mM analyte (10.7 mM caffeine or 11.8 mM terbutaline) in acceptor buffer was added above the PAMPA barrier. The tube was immediately placed into the spectrometer, and acquisition started. Slice-selective ^1^H spectra of the donor and acceptor chambers were continuously acquired for 24–48 h using the 1D slicesel_zgse (Figure S1A) pulse program with 32 scans and offset of +9 and −9 mm, respectively, with a total acquisition time of 4 min. The sampling interval was set to one spectrum every 20 min until the end of data collection.

The analyte concentration was quantified relative to the internal TMSP reference, and the apparent permeability (*P*_app_) was calculated according to eq 1.^16^

1 $$P_{\mathrm{app}}=\frac{dQ}{dt}\times \frac{1}{AC_{0}}$$

where d*Q*/d*t* is the change in analyte concentration over time, *A* is the area of the membrane, and *C*_0_ is the initial analyte concentration. d*Q*/d*t* was extracted from the analyte accumulating in the acceptor chamber, and the slope of the linear buildup was used to determine *P*_app_. The area, *A*, of the barrier used in the experiments was 0.03 cm^2^.

### pH Titration

To control the pH over a wide range, four pH reporters were used: Tris, formate, acetic acid, and imidazole. A stock solution was prepared containing 0.5 mM Tris, 3 mM ammonium formate, 3 mM imidazole, 3 mM acetic acid, 2.1 mM DSS, and 5% D_2_O in deionized water. pH calibration curves were obtained by adjusting the pH of the pH reporter stock solution (pH 2–12) across a series of discrete samples. ^1^H NMR spectra of each sample were recorded at 298 K using a standard zgeseq sequence available in the standard Bruker package. ^1^H chemical shifts were plotted as a function of pH, enabling extraction of the reporter p*K*_a_.

Titrations were performed as a one-shot experiment, meaning that the sample was fully prepared upfront and all data were subsequently acquired in automation without any further sample handling. First, 600 μL of 5 mM pyridine diluted in a pH reporter stock solution was added to a standard 5 mm NMR tube. The acceptor solution was acidified with 20 μL of 1 M HCl to ensure an acidic pH in the acceptor chamber. The insert disk was fitted with a 3.5 kDa Spectra/Por cutoff dialysis membrane. The insert was positioned in the tube high above the NMR detection volume to ensure minimal spectral interference. To begin the experiment, a donor solution of 60 μL of 1 M NaOH was added to the tube above the insert, the tube was immediately transferred to the spectrometer, and slice-selective ^1^H NMR spectra were continuously collected over 1–2 h. Spectra were recorded as 0.1 mm slices of the acceptor volume using the 1D slicesel_zges pulse program (Figure S1B) with 16 scans and an offset of 0 mm, with a total acquisition time per time point of 32 s.

The p*K*_a_ of the molecule of interest was finally determined by plotting its chemical shift as a function of the solution pH. The pH of each spectrum was determined using the pre-established calibration curves of the chemical shifts of the reporter molecules as a function of pH. The protonated (HA) and deprotonated (A^–^) states of each compound are in fast exchange with one another on the NMR time scale, resulting in one NMR signal per nucleus with a chemical shift (δ) corresponding to the weighted average of the HA and A^–^ states. Plotting the acquired δ_obs_ against pH results in a sigmoidal curve with p*K*_a_ at the inflection point (eq 2).^17^ Titration data were then fitted with eq 2 to extract the p*K*_a_ value.

2 $$pK_{a}=pH-log\left(\frac{\delta_{obs}-\delta_{HA}}{\delta_{A}-\delta_{obs}}\right)$$

where δ_HA_ and δ_A_ correspond to the limiting chemical shifts of the compound in its fully protonated and deprotonated forms, respectively.

### Urea Titration

First, 0.33 mM alginate degrading protein AlgE4R (in 20 mM HEPES and 25 mM CaCl_2_ (pH 6.5)) was added to a 5 mm NMR tube as the acceptor solution. The insert was fitted with a 12–14 kDa Spectra/Por cutoff dialysis membrane and positioned in the middle of the detection volume. The solution above the insert consisted of 250 μL of an 8 M urea stock solution in 20 mM HEPES ad 25 mM CaCl_2_ (pH 6.5), and the tube was immediately placed in the spectrometer after the addition of urea. To monitor the diffusion of urea through the membrane, slice-selective ^1^H NMR using the 1D slicesel_zges pulse program (Figure S1B) with four scans was performed on the donor side and spectra were collected every 10–30 min for ∼20 h. The urea peak was then integrated for each spectrum, with the absolute integral of the peaks correlated with urea concentration and plotted against the time of each acquisition. The concentration in the acceptor volume was estimated assuming that equilibrium is achieved at the end of the titration, implying that the concentrations on both sides of the diffusion barrier are identical at the end point. This gives a theoretical value of the effective volume of the acceptor (which was not measured by weight in this acquisition), which in turn yields the concentration in the acceptor volume. To monitor the unfolding of AlgE4R, standard ^1^H–^15^N HSQC experiments were performed using the BEST sequence b_hsqcetf3gpsi with 32 scans and td (^15^N) set to 64 and spectra were collected every 10–30 min for ∼20 h. The HSQC spectra were analyzed qualitatively to assess the denaturation of AlgE4R.

For manual titration of urea, 3 mm Shigemi tubes were used, with 130 μL of AlgE4R. For each titration, 26 μL of 8 M urea was added to the sample for a total of six titrations (182 μL of 8 M urea in total). To qualitatively monitor the unfolding of AlgE4R, ^1^H–^15^N HSQC spectra were collected using the BEST sequence b_hsqcetf3gpsi with 32 scans and td (^15^N) set to 64.

## Results and Discussion

### Development

The path that led to our technical device for high-quality *in situ* NMR spectroscopy started with a 3D-printed prototype. The prototype was of a tube-in-a-tube design, intended to test the concept of dividing the NMR tube into a donor volume and an acceptor volume and monitoring transport between two compartments. Preliminary results indicated that the tube-in-a-tube approach worked well as a “slow-release” application, which is sufficient for some applications such as measuring permeability or setting up a diffusion-controlled pH titration. In cases in which the donor compartment does not need to be monitored, there is no need to position the donor inside the detection volume, thereby removing the need to acquire z-selected data in the first place. In applications in which it is important to monitor the donor and acceptor volumes simultaneously, however, the tube-in-a-tube type approach did not offer satisfactory spectral quality of the donor compartment. Several drawbacks are associated with such approaches, including the additional glass–air–glass interface and the smaller diameter donor volume resulting in reduced spectral quality and inconsistency between the signal coming from the donor and the acceptor volume. Allowing the acceptor liquid to be pressed up around the donor chamber somewhat improves the spectral quality by removing the air interface, but in this situation, the acceptor signal is mixed into the donor volume signal, which is undesirable. We decided to discontinue that line of development in favor of the improved design described below. A tube-in-a-tube approach to monitor permeability has since been studied by another group.^18^

### Technical and Design Details

To overcome the inherent limitations of the tube-in-a-tube approach, an insertion disk was instead developed (Figure 2) to fit into a standard 5 mm NMR tube with an inner diameter of approximately 4.2 mm. The insertion disk allows the positioning of a physical barrier at a height of choice inside the tube, which divides it into two compartments with identical radial properties. The full inner diameter of the tube is utilized and introduces no additional interfaces between the glass and solvent or between the air and glass, and it does not push any of the acceptor solution up around the edges of the donor solution. This dramatically improves the spectral quality, uniformity, and sensitivity compared to those of the prototype.

**Figure 2:**
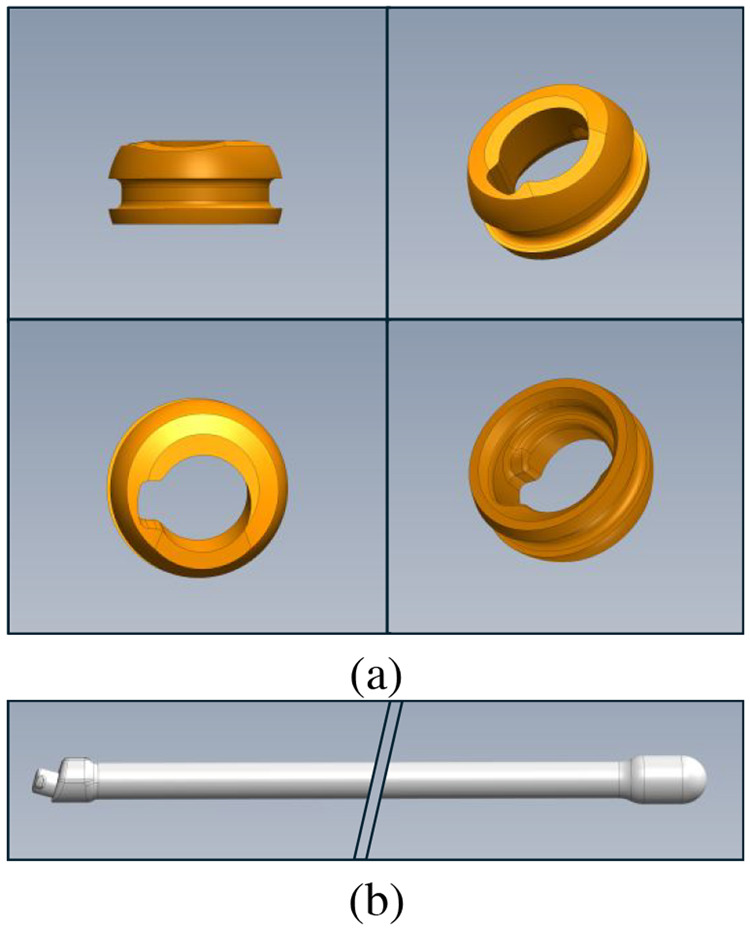
3D rendering of (a) the discoid insert and (b) the specially designed plunger tool for easy insertion and extraction of the disk into or from the NMR tube. The insert disk was manufactured by employing a high-precision machine workshop. Polyether ether ketone plastic (PEEK) was chosen as the insert disk material for its excellent thermal, chemical, and mechanical resistance properties. The plunger was made of stainless steel. The blueprint is provided in Scheme S1.

To allow for easy and reliable insertion and extraction of the disk, a specially designed plunger tool was developed. The tool inserts the disk at an angle to allow for the evacuation of trapped air pockets from underneath the disk. Once at the desired height, it can be rotated into place, leveling it and using friction against the inner glass walls of the tube to immobilize the disk and seal it (Figure 3). A gentle pull releases the plunger from the disk. A movie demonstrating the insertion and removal of the disk is available as Supporting Information.

**Figure 3:**
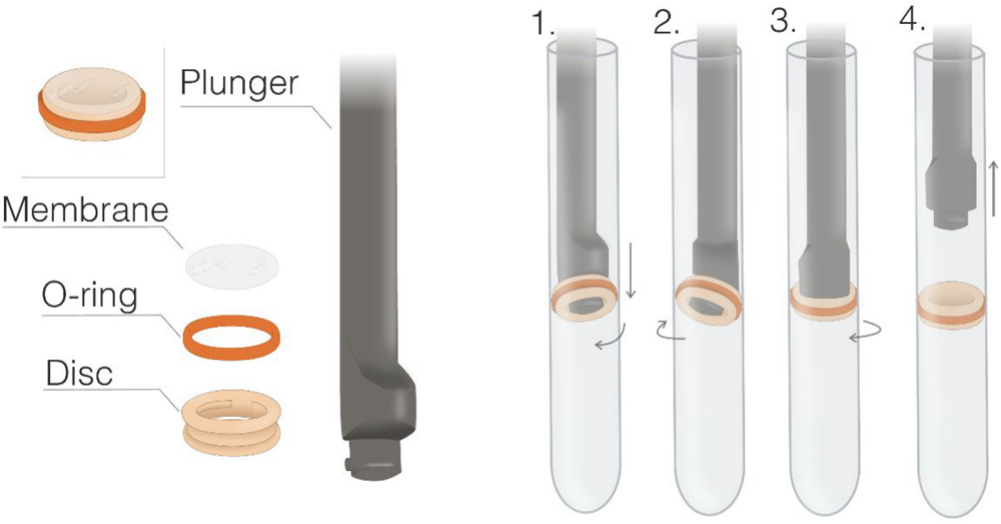
Cartoon figure showing how the insert disk is rotated into place using the designed plunger tool. The opposite motion is used to extract the disk from the tube. The Supporting Information includes a movie demonstration.

### Spectral Quality

The spectral quality was assessed by acquiring ^1^H z-profile images and an *in situ* NMR spectrum of 1 mm slices selected 8–10 mm above and below the barrier for the different setups, observing the Tris peak in a 1:9 H_2_O/D_2_O mixture (Figure 4). The results clearly showed that the disk insert gives good sensitivity from both sides of the barrier, whereas the tube-in-a-tube type approach inevitably sacrifices sensitivity from the donor volume as a direct result of the smaller volume inside the detection coil area. The disk also circumvents the problem with air bubble formation under the membrane upon insertion, which can otherwise require some rough handling to force the air bubble up around the edges that may damage barrier integrity. The presence of an insert or an inner tube inside the detection volume affects the line widths and absolute integrals, making it necessary to use an internal standard in the sample for accurate quantification in all cases. This affects all setups, but the insert disk caused less line broadening than the other approaches (summarized in Table S1).

**Figure 4:**
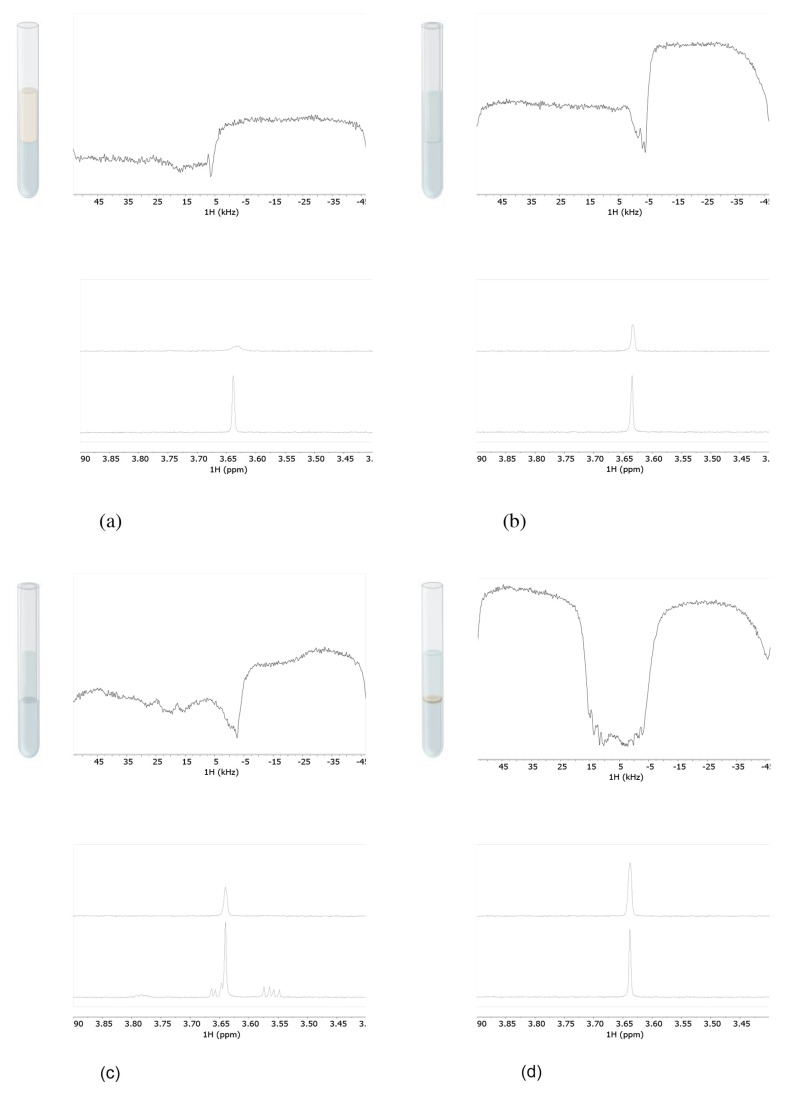
^1^H z-profile images (top panels) and *in situ* NMR spectra of 1 mm slices selected above and below the barrier (bottom panels) for (a) the 3D-printed tube-in-a-tube prototype with the donor liquid pressed up around the inner tube, (b) a commercial plastic NMR tube mounted in a high-precision glass tube with the donor liquid pressed up around the inner tube, (c) a membrane attached and less acceptor liquid surrounding the donor tube, and (d) the inNMR disk. All spectra show the Tris peak in a 1:9 H_2_O/D_2_O mixture and are plotted using an identical scale for comparability. The membrane used to create panel c comes stored in glycerol, giving rise to additional peaks between 3.5 and 3.7 ppm.

### Application

#### Permeability

The assessment of a compound’s permeability properties is a crucial part of the screening and preformulation processes in drug discovery and development. *In vitro* permeability assays, especially non-cell-based assays, are gaining interest as tools to obtain a reliable prediction of passive absorption properties without the disadvantages associated with cell- or tissue-based permeability profiling.^19^ The parallel artificial membrane permeability assay (PAMPA) is a simple and robust cell-free *in vitro* permeability method used in the pharmaceutical industry.^20^ Lipids are mixed in an organic solvent and applied to a membrane filter. The lipid-infused filter is then used to mimic different physiological membranes depending on the chosen lipid and chemical composition. Passive diffusion of compounds across the membrane from a donor chamber is measured by quantifying the concentration buildup in an acceptor chamber. The resulting apparent permeability (*P*_app_) is then used to estimate, for example, oral absorption or crossing of the blood–brain barrier depending on the type of barrier the artificial barrier is mimicking.^21^

To demonstrate the permeability application, we miniaturized the PAMPA experiment into a standard 5 mm NMR tube. The insert disk divides an NMR tube into a donor and acceptor chamber and facilitates a stable support for the lipid membrane. This enables simultaneous detection of the concentration buildup and decay in the acceptor and donor chambers, respectively, in real time. Traditional *in vitro* methods are based on physically moving a donor insert from one acceptor well to the next every 10–60 min on the lab bench and analyzing the acceptor solutions retrospectively. Analysis is therefore limited to a handful of collected data points of the acceptor solution. The use of the disk insert allows data to be acquired on both the donor and acceptor sides of the barrier in a single-shot NMR experiment with continuous acquisition, yielding unprecedented temporal accuracy.

As a proof of concept that the insert can be used to measure permeability, the *P*_app_ values of two model drugs, caffeine and terbutaline, with different water solubilities and absorption behavior, were measured (Figure 5). Terbutaline permeates the PAMPA barrier slower than caffeine and needed more than 40 h to reach 1 mM in the acceptor volume, while only 6 h was necessary for caffeine. The measured *P*_app_ values of caffeine and terbutaline were 11.0 and 1.9 × 10^–6^ cm/s, respectively. Many types of PAMPA exist utilizing different types, concentrations, and combinations of lipids and solvents. It should be noted that the absolute permeability values are highly dependent on the method, even across PAMPA variants, and *P*_app_ can vary greatly for a given compound. For example, there are literature values in the range of 0.6–10 × 10^–6^ cm/s for caffeine in PAMPA models. A similar PAMPA utilizing a type and a concentration of lipids and solvent comparable to those we have used has reported *P*_app_ values of 2.1 × 10^–6^ and 0.2 × 10^–6^ cm/s for caffeine and terbutaline, respectively.^22^ Significantly for the proof of concept, the insert can be used to measure the relative *P*_app_ between compounds, as demonstrated by the *P*_app_ of caffeine being an order of magnitude higher than that of terbutaline. The absolute difference between the acquired values and the literature values could be attributed to the preparation of the PAMPA barrier in the insert being different from the literature preparations in larger wells, differences in lipid composition, pH, and uncertainty in the effective radius of the membrane.

**Figure 5:**
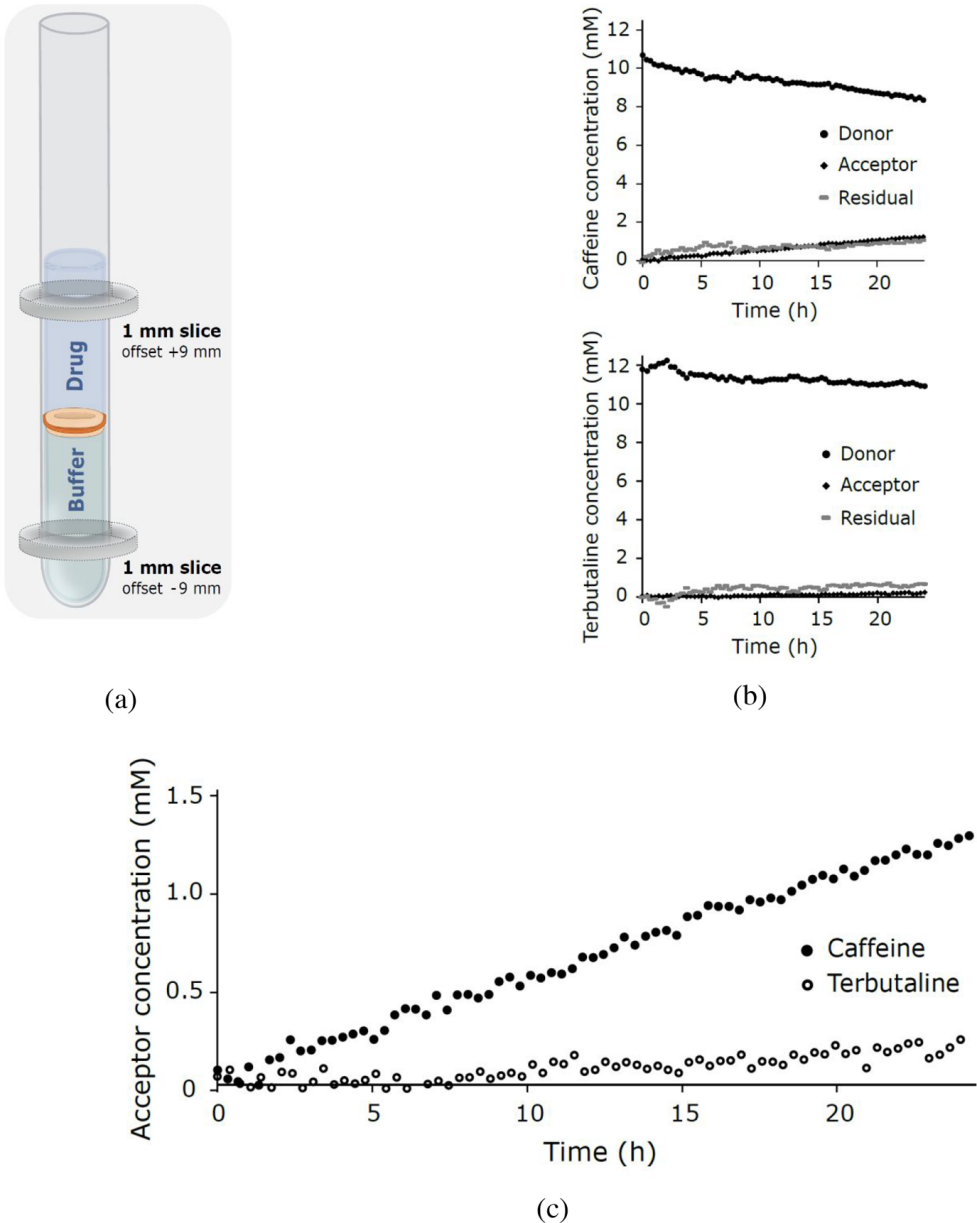
Proof of concept that permeability measurements can be acquired in a one-shot *in situ* experiment. (a) Experimental setup with the PAMPA barrier placed in the center of the detection volume. The drug is added in the donor volume. Spectra of the donor and acceptor volume are continuously recorded, allowing the drug concentration to be measured and plotted over time. (b) Acceptor and donor concentrations of caffeine and terbutaline, respectively, as well as the residual, defined as [initial] – [donor] – [acceptor]. (c) Comparison of their permeability in the acceptor.

In addition to measuring the concentration buildup in the acceptor in real time, the insert further allows for interleaved measurement of the donor concentration decay. This makes it straightforward to plot the residual between the donor decay and acceptor buildup, revealing any retention in the barrier. In our example, there was very little residual, indicating that all of the drug signal that disappears from the donor side appears as a detectable signal on the acceptor side.

These results demonstrate that permeability type experiments can be performed at an incredibly high time resolution. If one could overcome the difficulties with shimming *in situ* samples in full automation, then one could increase the throughput by analyzing more than 10 samples in parallel using interleaved acquisition in automation with a sample changer. The main strength of the insert disk approach is, however, the high time resolution, and the most interesting applications of the technique are probably to accurately sample dynamic processes in permeability related to thresholds, cooperativity, or release from nanoparticles.

#### pH Titration

The acid dissociation constant (p*K*_a_) is an important physicochemical constant usually obtained by titration, which can be both slow and labor-intensive as it requires the preparation and analysis of a series of solutions with graduated pH values. Efforts to develop more efficient methods have therefore been made. An innovative method by Wallace et al. was based on placing a solid acidic salt at the bottom of the NMR tube, which created a pH gradient as it dissolved.^23^ This allowed for the acquisition of a full titration curve from a series of spatially encoded 1D experiments, provided that a calibration curve for pH indicators was established beforehand (Figure 6a). However, the method depends on the creation of a stable gradient, which requires a long preparation time (up to 24 h) and could potentially be disturbed by automated sample handling.

**Figure 6:**
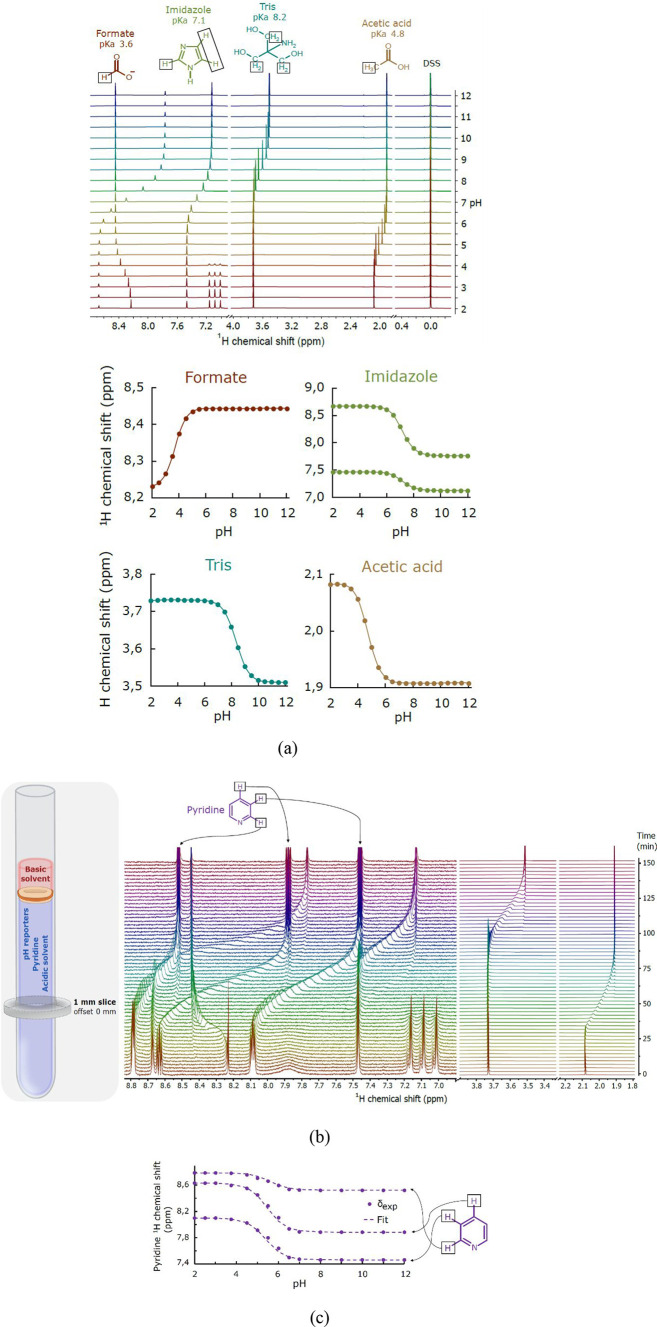
Proof of concept that pH titration can be monitored in a one-shot experiment using reporter molecules. (a) Reference titration curves of four different molecules sensitive to pH with different p*K*_a_ values: formate, imidazole, Tris, and acetic acid. (b) Experimental setup (left) with the diffusion barrier placed well above the detection volume. The response of the sample is monitored by a 1 mm slice to reduce the effect of any concentration gradients. In the acceptor volume, pyridine was added to the pH reporter molecules in order to measure its p*K*_a_. Results of a typical diffusion-controlled pH titration experiment (right). (c) Resulting pH curves for the three protons of the tested compound, pyridine.

A related but different approach to measuring p*K*_a_ can be taken using the NMR insert disk. Instead of creating a stable gradient, the titration is achieved by the slow release of a basic solution from the donor compartment into the acceptor compartment to create a dynamic pH gradient while spectra are being recorded continuously. The disk carrying a diffusion barrier is best placed well above the detection volume not to interfere with detection. The acidic (acceptor) and basic (donor) solutions are thus separated, and over time, the basic solution will diffuse into the acidic detection volume in a controlled manner. The analyte’s chemical shift response to the increasingly basic environment is then acquired together with the reporter molecules that track the local pH in the selected slice of the sample. The NMR insert disk thus offers an alternative way to acquire a pH titration curve that requires neither any sample preconditioning nor any repeated sample handling during the experiment.

The disk allows the measurement of a titration curve with a steady increase in pH over time in the acceptor volume (Figure 6b). The resulting diffusion-controlled pH titration enabled us to probe several hundred different pH environments per curve, which would be unviable in a conventional titration experiment that requires manual sample handling.

The time resolution can be adjusted by varying either the acquisition time or the diffusion speed. The diffusion time could be varied between 30 min and 11 h by modifying the pore size of the dialysis membrane (i.e., 3.5 or 12–14 kDa), the quantities of HCl and NaOH in the acceptor and donor volumes, respectively, and the relative densities between the two volumes (i.e., by adding KCl salt to one volume or the other).

The optimized experimental conditions used herein used a diffusion time of 90 min, striking a good compromise among experiment time, curve resolution, and spectral quality.

Analysis of the entire tube volume revealed substantial line broadening, indicating the presence of a weak concentration gradient in the length of the tube because of slow diffusion within the acceptor compartment. Selecting thinner slices improved the spectral quality, which was optimized to a 1–2 mm slice to achieve satisfactory sensitivity and line widths. However, for each species, two chemical shifts (one major and one less intense) were observed, indicating the presence of two different conditions across the width of the detected slice. It was confirmed using colored pH indicators (see Figure S2) that NaOH tends to diffuse along the walls inside the tube before homogenizing toward the center. Work is in progress to optimize the homogeneity.

The spectral quality was nevertheless sufficient to determine the pH accurately and measure the p*K*_a_ of an example molecule, pyridine. An average p*K*_a_ of 5.49 ± 0.12 was obtained from the three pyridine signals, which is in the range of reported literature values of ∼5.3 in water and ∼5.7 in D_2_O at 20–25 °C.^24^

Using diffusion to control a titration is the most straightforward in cases in which there are internal reporter molecules within the same detection slice as that of the analyte of interest. The analyte will therefore experience conditions identical to those of the reporters, making the diffusion-controlled pH titration demonstrated herein robust against any concentration gradients along the length of the NMR tube. The disk insert thus enables p*K*_a_ determination by NMR spectroscopy, which has traditionally required a labor-intensive day by a chemist, to be performed in 90 min without any sample handling after the experiment has been started.

#### Ligand Titration/Protein Unfolding

Understanding how biomolecules respond to interactions with target molecules or changes in their environment is an integral part of both specific industrial applications such as drug discovery and fundamental research of biological function. NMR is a powerful technique for such studies because it can simultaneously provide information about both the characteristics and sites of interaction. The most common way of studying interactions by NMR is by titrating a ligand (i.e., a drug to a target) or an environmental factor (i.e., metal ions, pH, or denaturing agent) into a fixed concentration of the ^15^N-labeled biomolecule. The increased level of binding can be detected as chemical shift perturbations, line broadening, or the appearance of new signals. Both ligand and protein experiments can be used to gain insight into binding dynamics, kinetics, and affinity.^25^

As a proof of concept, the NMR insert disk was used to set up a continuous titration of a denaturing agent into a protein solution, without the need for manual sample handling. The application relies on a known quantity of ligand being added to the target while detecting the response. The insert disk that allows simultaneous monitoring of the decay of the ligand in the donor chamber as well as the buildup of the effect in the acceptor chamber is therefore enabling in this sense, since the amount of added ligand cannot be reliably quantified in the acceptor chamber directly. This is due to the fact that the ligand protons will be in the same concentration regime as the target and be masked by the protein and the signals even if they could be separately detected would not be quantitative if the ligand is undergoing binding/unbinding exchange on the NMR time scale, which is very common in milli- to micromolar binding commonly seen for native ligands and screening fragments.

A simple protein unfolding experiment was set up by adding 0.33 mM ^15^N-labeled AlgE4R enzyme to the acceptor chamber and an 8 M urea solution to the donor chamber. The controlled diffusion induces a gradual unfolding process of the enzyme that can be observed by ^15^N HSQC. The gradual increase in denaturant concentration by diffusion allows approximately 10 times more experiments (*n* ∼ 100) to be performed than what is practical in a manual titration experiment (*n* ∼ 10) over the course of the experiment, resulting in a high resolution of the time/concentration dimension. This could allow detection of intermediate states that might be missed in a manual stepwise titration if too few titration steps are recorded. The experimental setup and the resulting gradual denaturation of the AlgE4R enzyme are displayed in Figure 7.

**Figure 7:**
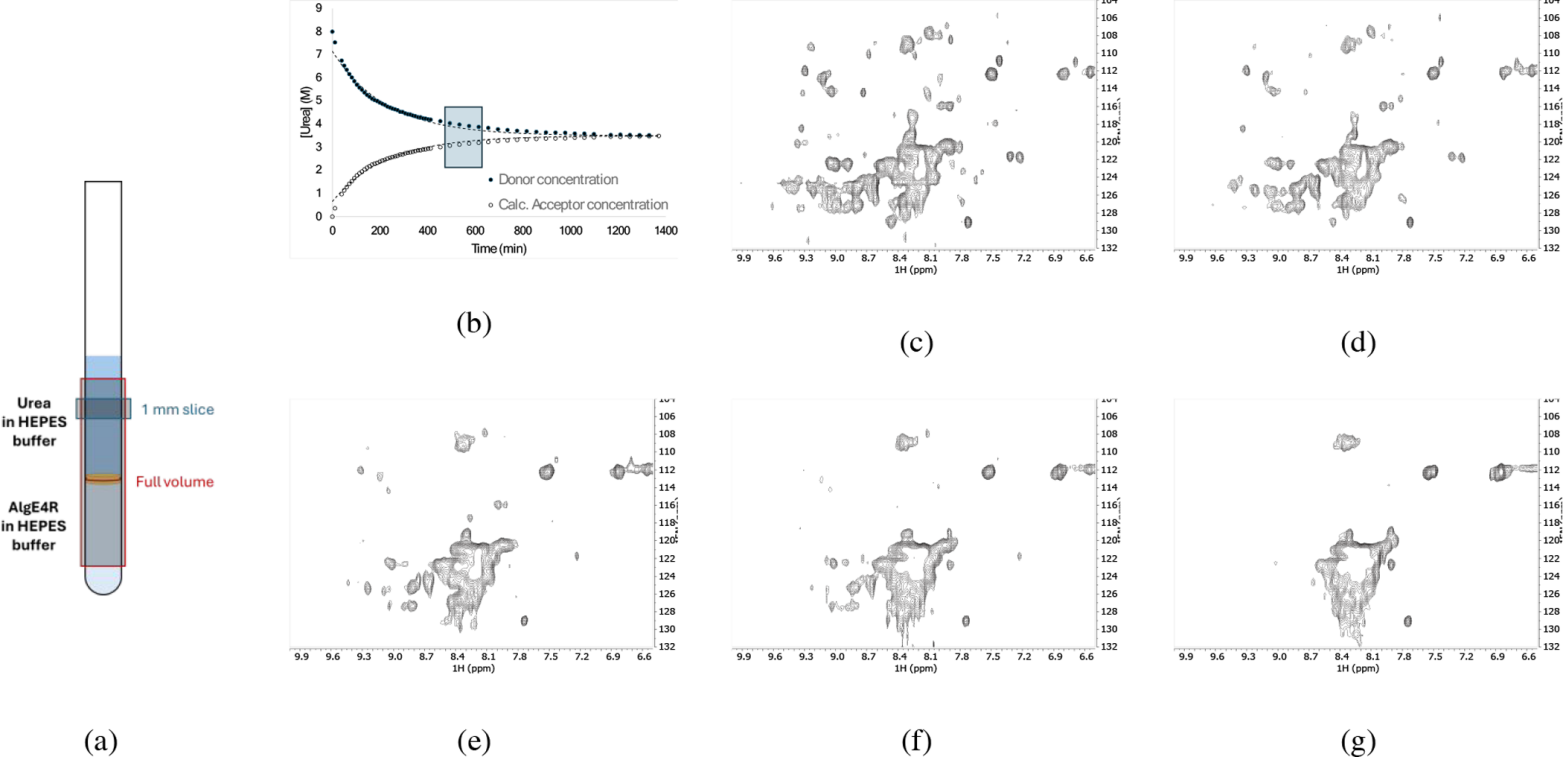
Proof of concept that the inNMR insert can be used to monitor a titration experiment in a one-shot overnight experiment. (a) Experimental setup, placing the insert in the middle of the detection volume. (b) Slice-selected 1D spectra are used to monitor the decay of the donor urea stock and plotted together with the calculated concentration in the acceptor volume. The denaturation takes place during the time frame of the highlighted box. (c–g) ^15^N HSQC spectra are acquired to monitor the response of the protein as a function of diffusion time. Representative 2D spectra during the highlighted time frame are presented.

A manual urea denaturation of AlgE4R was first performed, in which the protein fully unfolded between two data points: 3.0 and 3.5 M urea (Figure S3). The apparent denaturation concentration point in the diffusion-controlled experiment was in the range of 3.2–3.3 M urea (Figure 7b–g). This effectively demonstrates the concept that the insert technology can generate a very high-resolution titration without any sample handling during the experiment. The method will be further refined by measuring the response of the protein in the acceptor volume with SOFAST/BEST type slice-selective ^15^N HSQC to match the slice-selective 1D spectra that monitor ligand decay. This is expected to improve the time resolution, absolute accuracy, and spectral quality. Slice-selective HSQC and HMQC experiments to quickly characterize heterogeneous samples^26^ or to acquire fast spectra of homogeneous samples^27^ have been reported in the literature.

## Conclusions

We have developed a discoidal insert for standard NMR tubes (also compatible with 4 in. SampleJet tubes in automation) that enables *in situ* transport-based experiments by dividing the NMR tube into two chambers separated by a semipermeable membrane. The disk insert has improved spectral quality and reproducibility over earlier generation tube-in-a-tube approaches. The versatility of the insert was demonstrated by three potential applications (drug permeability studies using the flexible PAMPA system to assay drug permeability across *in vitro* membrane models, pH titration to determine the p*K*_a_, and protein unfolding using urea), all in experiments in which the sample is prepared upfront and all data are acquired automatically without any additional sample handling.

## Supplementary Material





## Notes

A movie demonstrating the insertion and removal of the disk (MP4)
Blueprint, schematic pulse sequences, a diffusion dye experiment, a manual urea titration, and pulse sequences for slice-selective spin echo and slice-selective excitation sculpting (PDF)

## Notes

Raw NMR data are available at Zenodo data repository 10.5281/zenodo.18095685.

## ∇

Department of Biomedical Sciences, University of Copenhagen, Blegdamsvej 3B, København N., Denmark

## Abbreviations

NMR: nuclear magnetic resonance
HSQC: heteronuclear single-quantum coherence
PAMPA: parallel artificial membrane permeability assay
