# Supplementary material for: InNMR: Direct In Situ Studies of Transport Phenomena Enabled by an Innovative NMR-Tube Insert
Source: Anal Chem. 2026 May 25;98(22):16399–408. doi: 10.1021/acs.analchem.6c01297 (PMC13261619; doi:10.1021/acs.analchem.6c01297)
Supplement: Supplementary file 2 [file ac6c01297_si_002.pdf]

Supporting Information

InNMR: Direct *in situ* studies of transport phenomena enabled by an innovative NMR-tube insert

Amelie Frison,[a] *Marit M. Sørensen,*[a] *Kristoffer Prince,*[a] *Maksim Mayzel,*[b] *Finn. L. Aachmann,*[c] *Gøril Eide Flaten,*[a] *Philip Rainsford,*[d†] *Johan Isaksson*[a,d*]

a. Department of Pharmacy, UiT the Arctic University of Norway, Box 6050 Langnes, 9037 Tromsø, Norway

b. Bruker Switzerland AG, Industriestrasse 26, 8117 Fällanden, Switzerland

c. Department of Biotechnology and Food Science, Norwegian University of Science and Technology (NTNU), Sem Sæland 6/8, 7491 Trondheim, Norway

d. Department of Chemistry, UiT the Arctic University of Norway, Box 6050 Langnes, 9037 Tromsø, Norway

* To whom correspondence should be addressed. Email: johan.isaksson@uit.no

Table of contents

Scheme S1. Blueprints of the (a) plunger tool and (b) insert disk.

Figure S1. (a) The slicesel_zgse pulse sequence is based on a standard DPFGSE pulse sequence where a gradient is applied during the selective refocusing pulse (reburp) to make it slice selective. (b) The slicesel_zges pulse sequence consists of a slice selective excitation pulse (eburp) that replaces the initial 90° pulse in a standard excitation sculpting sequence.

Figure S2. Example of an eye observation of a diffusion-controlled pH titration experiment. In this experiment, a 12-14 kDa cut-off dialysis membrane has been used. Acceptor chambers have been filled with 600 µL of the pH reporters solution (Tris, formate, acetic acid and imidazole) and 15 µL of HCl 1M. Donnor chambers have been filled with 50, 75 and 100 µL of NaOH (1M) + KCl (100 mM) solution from left to right, respectively. A drop of universal colored pH indicators has been added into each chamber. Pictures were captured (a) 10 min, (b) 1h and (c) 1h40 after filling the donor solution. (d) and (e) represent zoomed-in sections of the boxes in pictures (b) and (c), respectively, where inhomogeneous pH is visible. Note that among the numerous experiments conducted, only the worst ones are shown here.

Figure S3. Manually performed Urea denaturation of ^{15}N-labelled AlgE4R protein, with the Urea concentrations of: (a) 0 M, (b) 1.0 M (c) 1.5 M (d) 2.0 M (e) 2.5 M (f) 3.0 M.

Table S1. Summary of the half-height linewidths of the tris peak in H_2O:D_2O 1:9 for the four different setups evaluated in Figure 4. For reference, the linewidth of the tris peak without any insert in a standard proton spectrum was 1.8 Hz.

Pulse sequence S1. Slice selective spin echo

Pulse sequence S2. Slice selective excitation sculpting

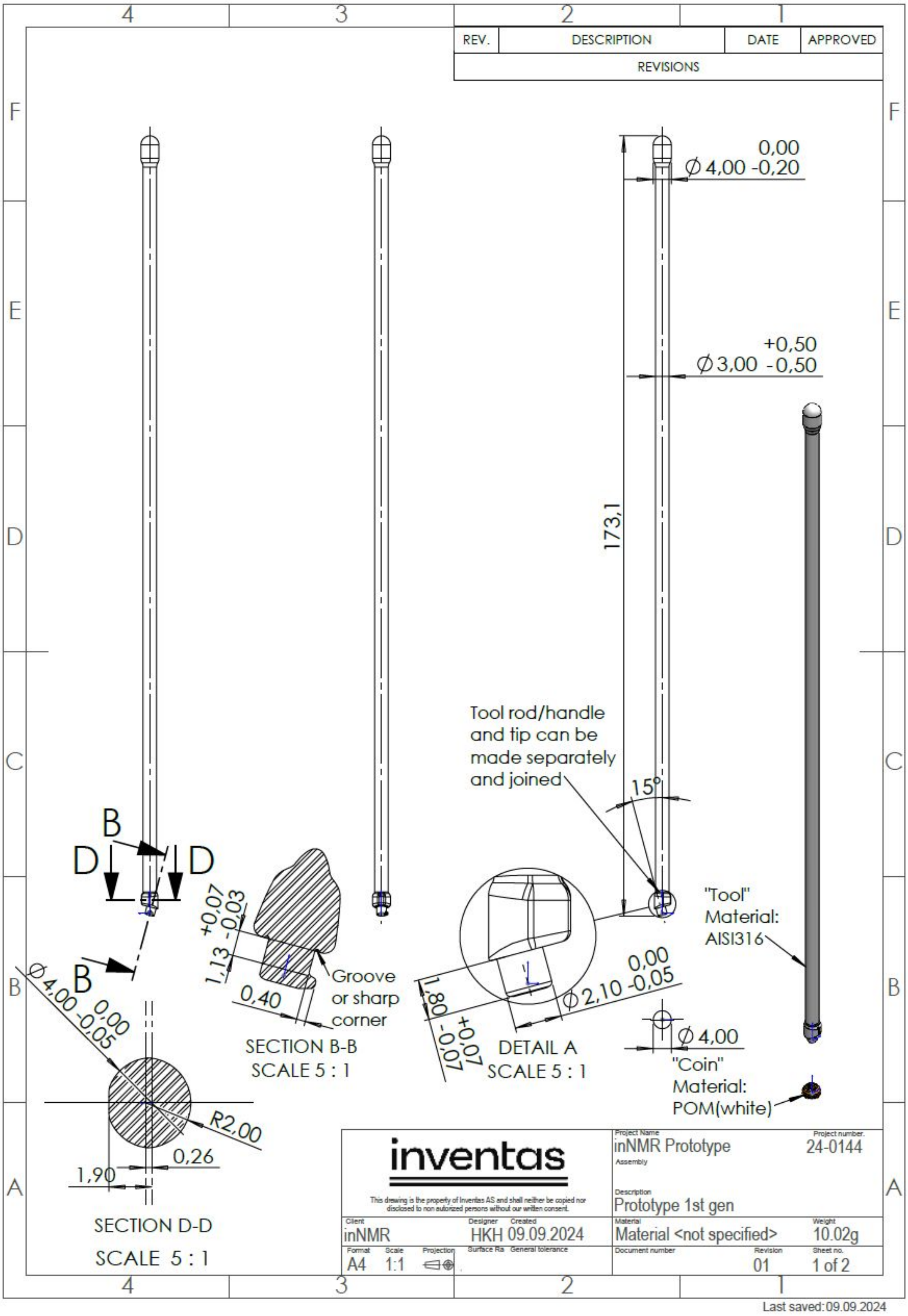
REV.
DESCRIPTION
DATE
APPROVED
REVISIONS
0,00
Ø 4,00 -0,20
+0,50
Ø 3,00 -0,50
173,1
Tool rod/handle and tip can be made separately and joined
15°
"Tool" Material: AISI316
Ø 4,00
"Coin" Material: POM(white)
B
D
D
B
+0,07
1,13 -0,03
0,40
Groove or sharp corner
SECTION B-B
SCALE 5 : 1
0,00
Ø 2,10 -0,05
1,80 +0,07 -0,07
DETAIL A
SCALE 5 : 1
0,00
Ø 4,00 -0,05
R2,00
0,26
1,90
SECTION D-D
SCALE 5 : 1
inventas
This drawing is the property of Inventas AS and shall neither be copied nor disclosed to non autorized persons without our written consent.
Project Name
inNMR Prototype
Project number.
24-0144
Assembly
Description
Prototype 1st gen
Client
inNMR
Designer
HKH
Created
09.09.2024
Material
Material <not specified>
Weight
10.02g
Format
A4
Scale
1:1
Projection
Surface Ra
General tolerance
Document number
Revision
01
Sheet no.
1 of 2
Last saved: 09.09.2024

(a)

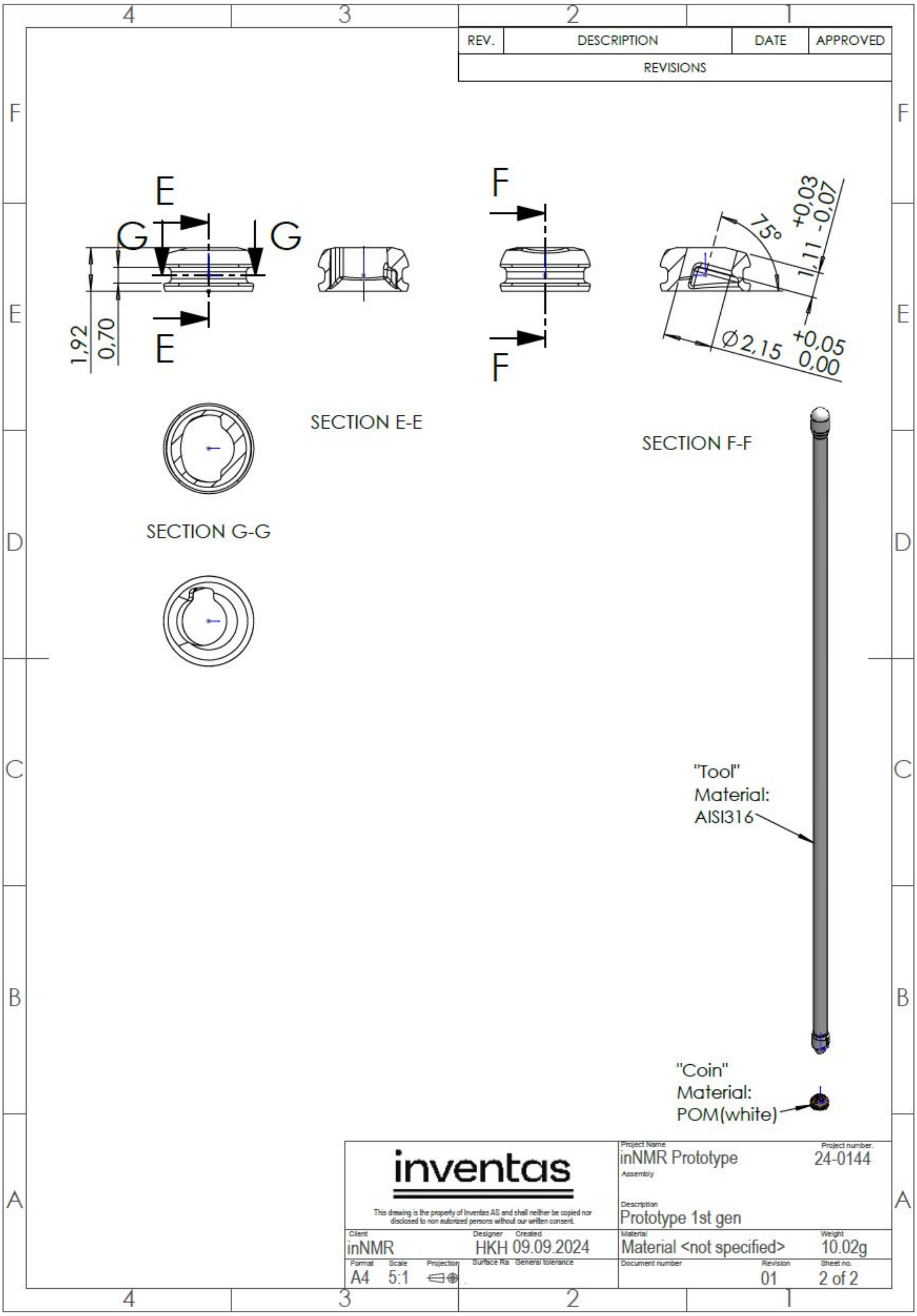

(b)

Scheme S1. Blueprints of the (a) plunger tool and (b) insert disk.

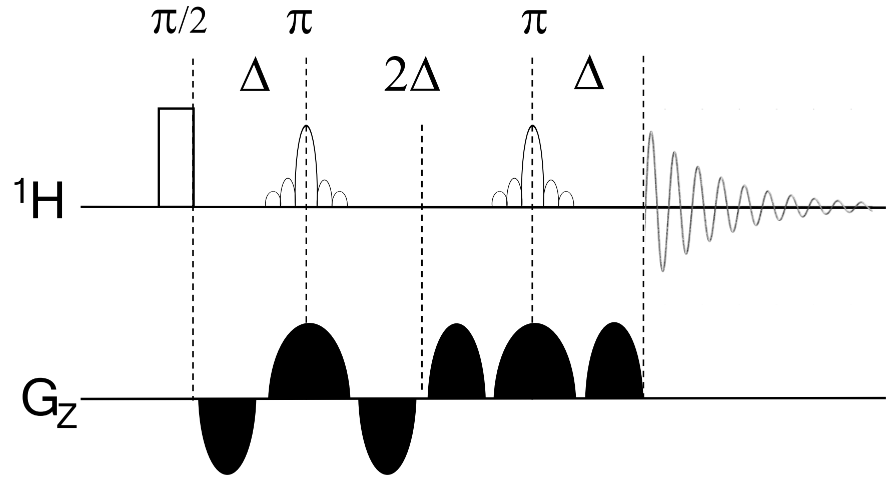

(a)

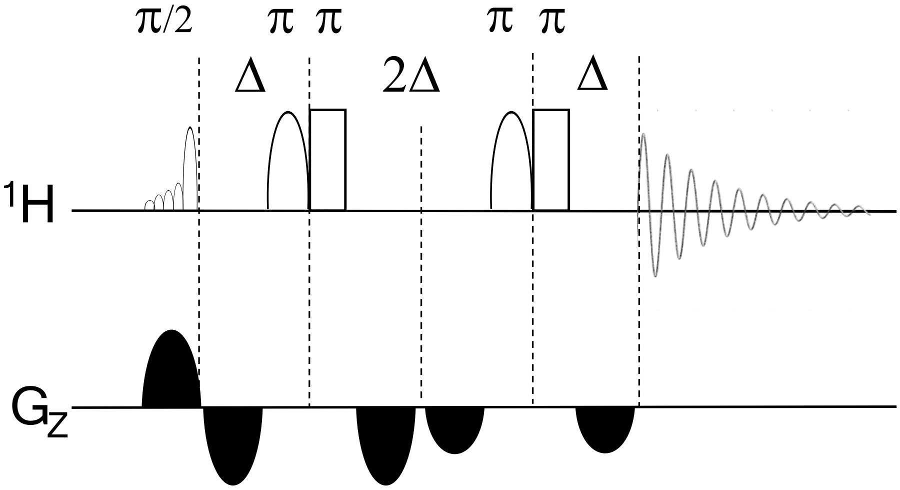

(b)

Figure S1. (a) The slicesel_zgse pulse sequence is based on a standard DPFGSE pulse sequence where a gradient is applied during the selective refocusing pulse (reburp) to make it slice selective. (b) The slicesel_zges pulse sequence consists of a slice selective excitation pulse (eburp) that replaces the initial 90° pulse in a standard excitation sculpting sequence.

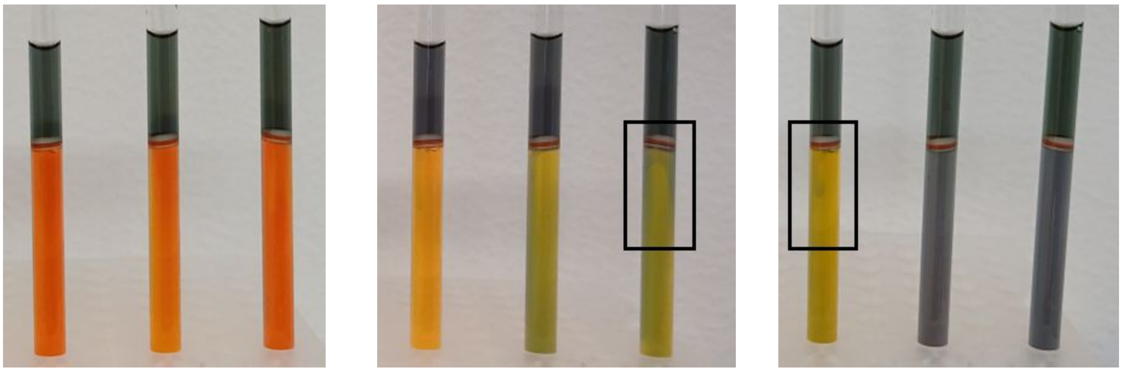

(a) (b) (c)

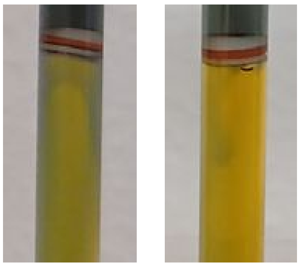

(d) (e)

Figure S2. Example of an eye observation of a diffusion-controlled pH titration experiment. In this experiment, a 12-14 kDa cut-off dialysis membrane has been used. Acceptor chambers have been filled with 600 µL of the pH reporters solution (Tris, formate, acetic acid and imidazole) and 15 µL of HCl 1M. Donnor chambers have been filled with 50, 75 and 100 µL of NaOH (1M) + KCl (100 mM) solution from left to right, respectively. A drop of universal colored pH indicators has been added into each chamber. Pictures were captured (a) 10 min, (b) 1h and (c) 1h40 after filling the donor solution. (d) and (e) represent zoomed-in sections of the boxes in pictures (b) and (c), respectively, where inhomogeneous pH is visible. Note that among the numerous experiments conducted, only the worst ones are shown here.

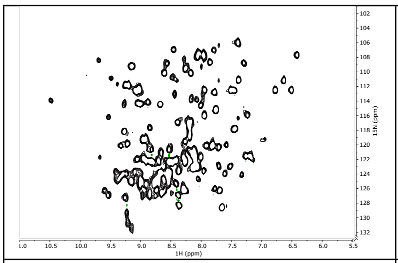	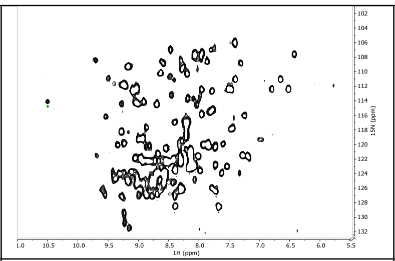	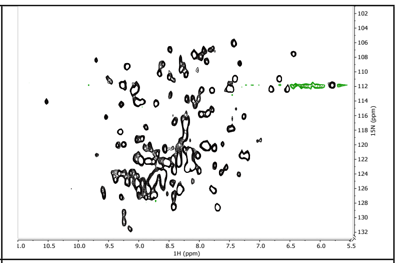
(a)	(b)	(c)
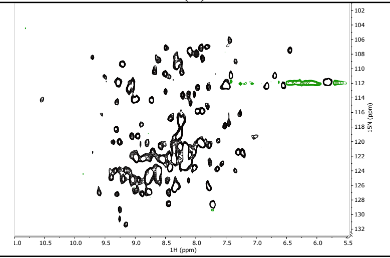	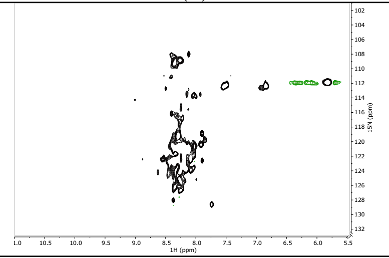	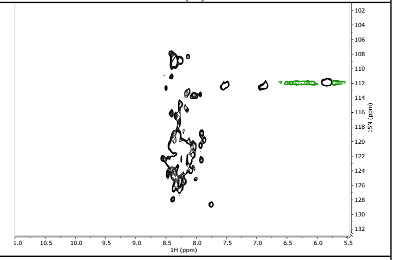
(d)	(e)	(f)

Figure S3. Manually performed Urea denaturation of ^{15}N-labelled AlgE4R protein, with the Urea concentrations of: (a) 0 M, (b) 1.0 M (c) 1.5 M (d) 2.0 M (e) 2.5 M (f) 3.0 M.

Table S1. Summary of the half-height linewidths of the tris peak in H_2O:D_2O 1:9 for the four different setups evaluated in Figure 4. For reference, the linewidth of the tris peak without any insert in a standard proton spectrum was 1.8 Hz.

	Half-height linewidth (Hz)	
Setup	High position, +8 mm	Low position, -8 mm
3D printed tube (a)	4.4	2.5
Tube w/o membrane (b)	3.8	2.5
Tube w/ membrane (c)	3.8	2.3
inNMR disk (d)	3.3	1.8

Pulse sequence S1. Slice selective spin echo

```
;slice selective 1D spin echo with gradients
;   using selective refocussing with a shaped pulse
;
;$CLASS=HighRes
;$DIM=1D
;$TYPE=
;$SUBTYPE=
;$COMMENT=


#include <Avance.incl>
#include <Grad.incl>
#include <De.incl>
#include <Delay.incl>

"d11=30m"
"d21=250u"
"gpnam0='RECT.1'"
;cnst57: bandwidth of the selective pulse [Hz]
;cnst58: spatial position of the slice in Z [cm]
;cnst59: freq span over entire coil y*G*z [Hz]
;cnst60: 100% gradient strength [~56.8 G/cm]
;cnst61: z-coil length [~2 cm]
;cnst62: slice thickness [cm]
;cnst63: 1H gyromagnetic ratio [Hz/G]
;"cnst60=63.4" ; TCI
;"cnst60=51"
"cnst63=4257.7"
;sw=gpz*y*G*z; gp=sw/(y*G*z)
"gpz0=100*(1e6/(2*dw))/(cnst63*cnst60*cnst62)" ; gradient strength enough to encode SW over
the slice width
"cnst59=gpz0*cnst63*cnst60*cnst61" ; frequency range encoded over the whole gradient coil
"spnam2='Reburp.1000'"
"p12=bwfac2*dw*2" ; shape covers the whole SW
"cnst57=1e6*bwfac2/p12"
"spoffs2=0.5*cnst58*cnst59"; spatial position of the slice in Z [cm]
"spw2=plw1*pow(p1*totrot2/(p12*90*integfac2),2)"
"spoal2=0.5"

"TAU1=de"
"TAU2=p0*2/PI" ;tan((p0/p1)*(PI/4))*p1*2/PI"
"d12=4u+cnst59*0+cnst57*0"
baseopt_echo
"acqt0=0"

1 ze
```

```
2 30m BLKGRAD
#ifdef presat
  4u pl9:f1
  d1 cw:f1
  4u do:f1
  50u UNBLKGRAD
  p19:gp3
  d16
#else
  d1
  50u UNBLKGRAD
#endif
  4u pl1:f1

  (p0 ph1):f1
  TAU1
  p19:gp1
  d16
  (center (p12:sp2 ph1):f1 (p12:gp0)) ; slice-selective refocusing
  p19:gp1
  d16
#ifndef singleEcho
  p19:gp2
  d16
  (center (p12:sp2 ph1):f1 (p12:gp0)) ; slice-selective refocusing
  p19:gp2
  d16
#endif
  TAU2
  go=2 ph31
  30m mc #0 to 2 F0(zd)

  4u BLKGRAD
  d12
exit


ph1=0
ph2=1
ph30=0
ph31=0


;pl0 : 0W
;pl1 : f1 channel - power level for pulse (default)

;p1 : f1 channel -  90 degree high power pulse
;p12: f1 channel - 180 degree shaped pulse
;p16: homospoil/gradient pulse                            [1 msec]
;d1 : relaxation delay; 1-5 * T1
;d15: variable echo time used for phase encoding [5-25ms]
;d21: read gradient stab. delay [ 250us]
;ns: 1
;ds: 0

;for z-only gradients:
;gpz0: 11%

;use gradient files:
;gpnam0: RECT.1

;gpnam1: SMSQ10.100
;gpnam2: SMSQ10.100
;gpnam3: SMSQ10.100
;gpz1: -17
;gpz2: 7
;gpz3: 13
```

Pulse sequence S2. Slice selective excitation sculpting

```
;slice selective 1D water suppression using excitation sculpting
;   using selective refocussing with a shaped pulse
;$CLASS=HighRes
;$DIM=1D
;$TYPE=
;$SUBTYPE=
```

```
;$COMMENT=

prosol relations=<triple>

#include <Avance.incl>
#include <Grad.incl>
#include <Delay.incl>


"p2=p1*2"
"d12=20u"

"gpnam0='RECT.1'"
;cnst57: bandwidth of the selective pulse [Hz]
;cnst58: spatial position of the slice in Z [cm]
;cnst59: freq span over entire coil y*G*z [Hz]
;cnst60: 100% gradient strength [~56.8 G/cm]
;cnst61: z-coil length [~2 cm]
;cnst62: slice thickness [cm]
;cnst63: 1H gyromagnetic ratio [Hz/G]
;"cnst60=56.8" ; QCIF
"cnst60=63.2" ; CP-TCI, UiT
"cnst63=4257.7"

;sw=gpz*y*G*z; gp=sw/(y*G*z)
"gpz0=100*(1e6/(2*dw))/(cnst63*cnst60*cnst62)" ; gradient strength enough to encode SW over
the slice width
"cnst59=gpz0*cnst63*cnst60*cnst61" ; frequency range encoded over the whole gradient coil

"spnam28='Eburp2.1000'"
"p43=bwfac28*dw*2" ; shape covers the whole SW
"spoffs28=0.5*cnst58*cnst59"; spatial position of the slice in Z [cm]
"spw28=plw1*pow(p1*totrot1/(p43*90*integfac28),2)"
"spoal28=1"

"TAU=de"

"acqt0=0"
baseopt_echo


1 ze
2 30m
  d12 pl1:f1 BLKGRAD
  d1
  50u UNBLKGRAD
  (center (p43:sp28 ph1):f1 (p43:gp0)) ; slice-selective excitation

  p19:gp1
  d16
  (p12:sp1 ph2:r):f1
  4u
  (p2 ph3 pl1):f1
  4u
  p19:gp1
  d16
  TAU
  p19:gp2
  d16
  (p12:sp1 ph4:r):f1
  4u
  (p2 ph5 pl1):f1
  4u
  p19:gp2
  d16

  go=2 ph31
  30m mc #0 to 2 F0(zd)
  4u BLKGRAD
exit

ph1=0
ph2=0 1
ph3=2 3
ph4=0 0 1 1
ph5=2 2 3 3
ph31=0 2 2 0
```

```
; ph1=0
; ph2=0 1
; ph3=2 3
; ph4=0 0 1 1
; ph5=2 2 3 3
; ph6=1
; ph7=0
; ph8=2
; ph31=0 2 2 0


;pl1 : f1 channel - power level for pulse (default)
;sp1 : f1 channel - shaped pulse 180 degree
;p1 : f1 channel -  90 degree high power pulse
;p2 : f1 channel - 180 degree high power pulse
;p12: f1 channel - 180 degree shaped pulse (Squa100.1000)   [2 msec]
;p16: homospoil/gradient pulse
;d1 : relaxation delay; 1-5 * T1
;d12: delay for power switching                             [20 usec]
;d16: delay for homospoil/gradient recovery
;ns: 8 * n, total number of scans: NS * TD0
;ds: 4


;for z-only gradients:
;gpz1: -31%
;gpz2: -11%
;gpz3: 5%

;use gradient files:
;gpnam1: SMSQ10.100
;gpnam2: SMSQ10.100
;gpnam3: SMSQ10.100



;$Id:$
```
